# Extracellular vesicles derived from CD73 modified human umbilical cord mesenchymal stem cells ameliorate inflammation after spinal cord injury

**DOI:** 10.1186/s12951-021-01022-z

**Published:** 2021-09-08

**Authors:** Xiao Zhai, Kai Chen, Huan Yang, Bo Li, Tianjunke Zhou, Haojue Wang, Huipeng Zhou, Shaofeng Chen, Xiaoyi Zhou, Xiaozhao Wei, Yushu Bai, Ming Li

**Affiliations:** 1 Department of Orthopedics, Shanghai Changhai Hospital, Naval Medical University, Shanghai, 200433 China; 2 Basic Medicine College, Naval Medical University, Shanghai, 200433 China

**Keywords:** Spinal cord injury, CD73, Extracellular vesicles, Mesenchymal stem cell, Inflammation

## Abstract

**Background:**

Spinal cord injury (SCI) is an inflammatory condition, and excessive adenosine triphosphate (ATP) is released into the extracellular space, which can be catabolized into adenosine by CD73. Extracellular vesicles have been designed as nano drug carriers in many diseases. However, their impacts on delivery of CD73 after SCI are not yet known. We aimed to construct CD73 modified extracellular vesicles and explore the anti-inflammatory effects after SCI.

**Methods:**

CD73 engineered extracellular vesicles (CD73+ hucMSC-EVs) were firstly established, which were derived from human umbilical cord mesenchymal stem cells (hucMSCs) transduced by lentiviral vectors to upregulate the expression of CD73. Effects of CD73+ hucMSC-EVs on hydrolyzing ATP into adenosine were detected. The polarization of M2/M1 was verified by immunofluorescence. Furthermore, A2aR and A_2b_R inhibitors and A2bR knockdown cells were used to investigate the activated adenosine receptor. Biomarkers of microglia and levels of cAMP/PKA were also detected. Repetitively in vivo study, morphology staining, flow cytometry, cytokine analysis, and ELISA assay, were also applied for verifications.

**Results:**

CD73+ hucMSC-EVs reduced concentration of ATP and promoted the level of adenosine. In vitro experiments, CD73+ hucMSC-EVs increased macrophages/microglia M2:M1 polarization, activated adenosine 2b receptor (A2bR), and then promoted cAMP/PKA signaling pathway. In mice using model of thoracic spinal cord contusion injury, CD73+ hucMSC-EVs improved the functional recovery after SCI through decreasing the content of ATP in cerebrospinal fluid and improving the polarization from M1 to M2 phenotype. Thus, the cascaded pro-inflammatory cytokines were downregulated, such as TNF-α, IL-1β, and IL-6, while the anti-inflammatory cytokines were upregulated, such as IL-10 and IL-4.

**Conclusions:**

CD73+ hucMSC-EVs ameliorated inflammation after spinal cord injury by reducing extracellular ATP, promoting A2bR/cAMP/PKA pathway and M2/M1 polarization. CD73+ hucMSC-EVs might be promising nano drugs for clinical application in SCI therapy.

**Graphical Abstract:**

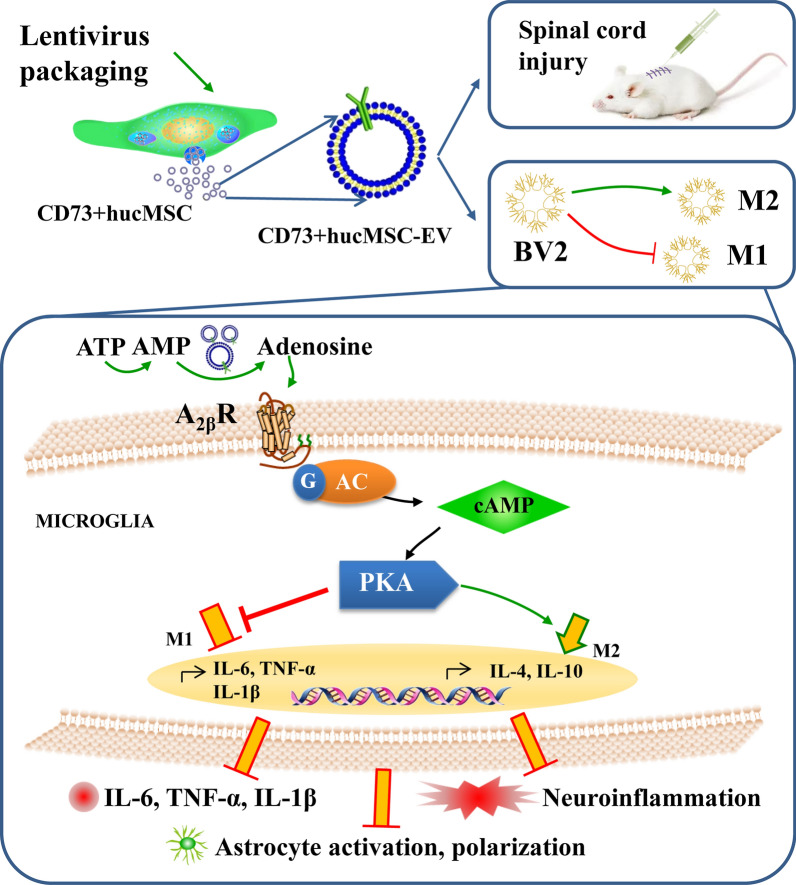

**Supplementary Information:**

The online version contains supplementary material available at 10.1186/s12951-021-01022-z.

## Introduction

Spinal cord injury (SCI) affects 12,000 individuals annually in the U.S., and current medicamentous and therapeutic possibilities after SCI are very limited [1]. It covers various types of damage to the neural cells of spinal cord, and initiates a complex secondary cascade effect with an elevated inflammatory state [2, 3]. After acute injury, macrophages constitute the first line of defense, can migrate to site of damage, secrete inflammatory cytokines, and phagocytose foreign debris [4, 5]. The infiltrating macrophages and in situ microglia reach their peaks on 3–7 days after SCI, and polarize to pro-inflammatory M1 subtype, which further secrete pro-inflammatory cytokines and lead to the secondary injury. Moreover, this microglia might then trigger A1 phenotype astrocyte proliferation and astrocyte scar formation, which is harmful for the surrounding microenvironment. On the other hand, macrophages and microglia can alternatively polarize into anti-inflammatory M2 phenotype and reduce inflammatory responses [6, 7]. Therefore, the balance between M1 and M2 subtypes may become potential targets for medication therapy in SCI [8].

Under inflammatory conditions of SCI, excessive adenosine triphosphate (ATP) is released from the cell cytoplasm into the extracellular space, amplifying inflammatory immune response through the recruitment and activation of immune cells [9, 10]. Extracellular ATP can be catabolized into adenosine monophosphate (AMP) by CD39 rapidly, but the conversion of AMP to adenosine is limited by CD73 (ecto-5′-nucleotidase), the rate-limiting ecto-enzyme in extracellular AMP hydrolysis [11]. CD73, as an extracellular nucleotide enzyme attached to the plasma membrane, may affect the remediation of purine nucleotides. However, its effect on SCI remains unknown due to the impermeability of large molecules crossing blood–brain barrier (BBB) and the possibility of tumorigenicity [12, 13].

By using the gene delivery systems, extracellular vesicles (EVs) derived from mesenchymal stem cells (MSC-EVs) can be applied wider as nanoparticle-based drug delivery systems [14]. Human umbilical cord derived MSCs (hucMSCs) can be harvested without any invasive operations, and have been shown to have more efficient proliferation, when compared to bone marrow-derived MSCs [15]. In addition, extracellular vesicles derived from human umbilical cord MSCs (hucMSC-EVs) not only possess major characteristics from hucMSCs, but also have low immunogenicity and nanoscale size that can easily permeate across the BBB [16]. As a result, hucMSC-EVs might be efficient drug carrier systems for the central nervous system, which can increase the efficacy of the drug and minimize the side effects [17].

In this study, mouse model of thoracic spinal cord contusion injury and vitro model using BV2 cells were applied to study neuroinflammation. Firstly, SCI models involve acute contusion [18] and chronic complete transection [19]. Spinal cord contusion accounts for the vast majority of clinical cases and the transection model could not objectively mimic clinical pathological changes of SCI, therefore we explore the effects after spinal cord contusion injury. And then, BV2 cell line is derived from immortalized murine neonatal microglia. It is the most frequently used as an in vitro microglial model, since it revealed similarities with microglial in the presence of LPS in transcriptomic and proteomic analyses [20].

Herein, we hypothesized that EVs derived from CD73 modified hucMSCs (CD73+ hucMSC-EVs) have a positive role in ameliorating SCI. Lentiviral vectors were used to transduce CD73 overexpressed hucMSCs, and EVs were isolated. Mice using model of thoracic spinal cord contusion injury and cells induced by LPS/IL-4 were treated with CD73+ hucMSC-EVs. Surprisingly, we found that CD73+hucMSC-EVs could reduce concentration of ATP and promote level of adenosine, attenuate the inflammation of spinal cord, upregulate macrophages/microglia M2:M1 polarization, via an adenosine/adenosine 2b receptor (A_2b_R)/cAMP/PKA signaling pathway. It suggested that CD73+hucMSC-EVs might have promising potential for clinical application in SCI therapy.

## Materials and methods

### Cell cultures

Umbilical cord tissue was harvested from informed consenting healthy mothers and approved by the hospital clinic ethics committee (institutional review board (IRB) No. 81701199). Under sterile conditions, the cord blood was washed and removed. Cords were chopped into 1 mm pieces and transferred into DMEM containing 10% FBS, 5%HS, 1% P/S(v/v), and collagenase II (1 g/L; Nanjing KeyGen Biotechnology Co., Ltd., Nanjing, China) at 37℃ in an incubator containing 5% CO_2_. Centrifuged and washed with PBS, non-adherent cells were removed. Cells were then plated into a culture flask at a density of 1 × 10^6^/mL and the culture medium was replaced every 3 days. After 10 days, cells reached 90% confluency and appeared colonies of fibroblast-like cells, and then they were digested in trypsin and passed into a new plate. HucMSCs were cultured in stem cell culture medium (Cyagen, Guangzhou, China) for further expansion.

BV2 cells, and astrocytes were cultured under complete medium (DMEM, Gibco, Carlsbad, CA, USA) with 10% Fetal Bovine Serum (FBS, Gibco, Carlsbad, CA, USA) and 1% penicillin streptomycin antibiotic (HyClone). BV2 cells cultured in the above medium were defined as M0 macrophages. M0 macrophages were undergone with LPS/interleukin 4 (IL-4) for 8 h to activate M1/M2 phase [21].

### Production of lentiviral vectors and transduction of hucMSCs

To produce lentiviral vectors, the pCDH lentiviral plasmid was used to make the virus in a class II biosafety laboratory. The pCDH-GFP vector (Asia-Vector Biotechnology Co., Ltd, Shanghai, China) was constructed based on CD73-overexpressed plasmid, which was prepared from a synthetic gene including the CD73 sequence. The human embryonic kidney 293 T (HEK-293T) cell line was transfected with the lentivirus and packaging plasmids using Lipofectamine 3000 (Thermo Fisher Scientific, Waltham, MA) [22].

To determine the minimum lethal concentration, hucMSCs were treated with puromycin. At concentrations > 20 µl/ml for 6 days, almost 100% hucMSCs were undergone apoptosis; therefore 20 µl/ml was selected as the optimal puromycin screening concentration.

For transduction, hucMSCs were prepared in 12-well plates with 3–5 × 10^5^ per well, and then were cultured to 70–80% confluency. In the next day, the prepared hucMSCs were transduced with lentiviral viruses in the presence of 10 μg/ml polyberene (Sigma, Germany). The medium was changed every 12 h. In addition, 20 µl/ml puromycin was added at 24 h and changed every 2 days. Finally, fluorescent microscope and light microscope were used to check the activity of harvested stable cell lines of CD73+hucMSCs.

### EVs isolation and characterization

The isolation of EVs was performed using ultracentrifugation and size exclusion chromatography as described previously [23]. The hucMSCs and CD73+hucMSCs added with MesenCult Stimulatory Supplements (STEMCELL Technologies, Vancouver, Canada) were ultracentrifuged at 100,000 g for 18 h using ultracentrifugation (MICROCL, Thermo Fisher Scientific, Inc., USA) to remove cell debris. The supernatant was filtered through a 0.22 μm pore filter (Millipore), and then was centrifuged at 3000 g for 30 min, 10,000 g for 30 min to remove cells and debris. Therefore, the supernatant was concentrated volume by ultrafiltration (100 kD, Millipore, USA) at 2000 g for 8min [18]. And then, EVs obtained from ultracentrifugation were loaded onto qEV Size Exclusion column (Izon Science, Christchurch, New Zealand) according to the instruction manual [24], which allowed to separate EVs based on the size into 16 fractions. The 16 fractions were concentrated by a vacuum centrifuge. Subsequently, the collected particles were washed with phosphatebuffered saline (PBS) to resuspend EVs and ultracentrifuged at 100,000 g for 70 min twice. All centrifugation was operated at 4 °C, and finally the solution was stored at − 80 °C or used immediately for experiments.

For extracellular vesicle identification, the morphology of purified EVs was identified by transmission electron microscopy (TEM, JEM-2100F, Japan Electronics Co., Ltd.). The size distribution and concentration were examined by nanoparticle tracking analysis (NTA, ZetaView, Particle Metrix Inc., German).

### Measurement of the ATP and AMP hydrolytic of EVs

EVs were repelleted by ultracentrifugation and resuspended in MES buffer (Sigma) to remove inorganic phosphate in PBS. And then, treated with 20 μM ATP or AMP (Sigma) in 500 μl of MES for 1 h, an indicated dose of 3, 10 or 30 μg/ml hucMSCs-EXs or CD73+hucMSC-EVs were added with or without 100 μM APCP (Santa Cruz Biotechnology, Santa Cruz, CA). The ATP Assay Kit (Beyotime, Shanghai, China) was used to test the concentrations of ATP, and SensoLyte MG Phosphate Assay Kit (Anaspec, USA) was applied to quantify phosphate produced by ATP or AMP. The Adenosine Assay Kit (BioVision, CA, USA) was employed for measuring concentrations of adenosine [25].

### Immunofluorescence

The cultured cells and each spinal cord slides were prepared for fluorescence microscopy by permeabilization for 5 min with 0.1% Triton- × 100, blocked with 5% BSA then incubated overnight with anti-iNOS (1:100; ab49999, Abcam Cambridge, MA, USA), anti-Arg1 (1:100; ab133543, Abcam Cambridge, MA, USA), anti-C3 (1:100; 21337–1-AP, ProteinTech, Manchester, UK), anti-S100A10 (1:100; 11250–1-AP, ProteinTech, Manchester, UK), or anti-GFAP (1:100; sc-33673, Santa Cruz Biotechnology, Santa Cruz, CA, USA). Cells were incubated with secondary antibody, Alexa Fluor 488 goat anti-mouse IgG (H + L) antibody/Alexa Fluor 594 horse anti-rabbit secondary antibody (all 1:1000; Jackson ImmunoResearch, West Grove, PA) for 1 h at room temperature. A DAPI solution was applied for 5 min for nuclear staining. Images were captured using a Leica TCA SP8 confocal laser scanning microscopy (Leica, Germany) [26].

### Western blot

Cells and EVs were lysed with ice-cold radioimmunoprecipitation assay buffer (Solarbio, Beijing, China) supplemented with a protease and phosphatase-inhibitor cocktail (Abcam, Cambridge, MA, USA), and the protein concentration was estimated using a BCA protein assay kit (Beyotime, P0010). And then, the protein samples were dissolved in SDS–polyacrylamide gel electrophoresis and transferred to nitrocellulose membranes. Blocked in locking buffer (TBST), it was incubated with primary antibodies (1:1000) at 4 °C overnight [27, 28]. Antibodies of CD73, CD9, CD63, CD81, TSG101, ALIX, calnexin, PKA, iNOS, and Arg1, were purchased from Abcam (Abcam, CA, USA). GAPDH antibody was purchased from Santa Cruz Biotechnology (Santa Cruz, CA, USA). After washing with TBST for three times, the membranes were incubated with horseradish peroxidase-conjugated secondary antibody (1:5000) and bands were developed using enhanced chemiluminescence. Image analysis was performed using Image J (NIH Image J system, Bethesda, MD).

### Quantitative real-time PCR (qRT-PCR) analysis

Total RNA was isolated using the TRIzol Reagent (Thermo Fisher Scientific, USA) following the manufacturer’s instructions. The RNA concentration was quantified by the NanoDrop ND-2000 (Thermo Fisher Scientific, USA). The SYBR Green reagent was used in qRT-PCR mRNA quantification. Corresponding primers are listed in Additional file 1: Table S1.

### Intracellular cAMP measurements

Intracellular cAMP was with the specific enzymeimmunoassay Biotrak (EIA) System (Amersham), following the manufacturer’s instructions. Briefly, 10^5^ BV2 cells were plated and incubated with 1 μM/mL LPS. Optical density was read at 450 nm and results were calculated [29].

### Transfection of shRNA

BV2 cells (15,000 cells/ml) were plated for 24 h, and then were incubated separately with 10^8^ MISSION® shRNA Lentiviral Transduction Particles for human A2b ADO receptors, as well as scrambled non-targeted shRNA (SHC002V) as control. The transfection was conducted according to manufacturer's recommendations, and then cells were selected by addition of puromycin (Sigma) at 2 µg/ml. The expression of A2bR was tested by quantitative PCR to verify its effect of knockdown [29].

### Animal model of spinal cord injury

Six-week-old male ICR mice (30–35 g) were purchased from SLAC Company (Shanghai, China). All mice were kept in the specific pathogen-free (SPF) laboratory, and the experimental procedures were approved by the Institutional Lab Animal Care and Use Committee.

Mice were anesthetized with pentobarbital sodium (50 mg/kg i.p.). Bilateral laminectomy of T8–T9 was performed to expose the spinal cord. A New York University Impactor was used to cause contusive SCI with a weight drop injury using a 10 g rod dropped at a height of 6.25 mm. After injury, the muscle and the skin were closed, and mice were placed in a temperature and humidity-controlled chamber. Manual bladder emptying was performed three times daily [30].

### Treatment of hucMSC derived EVs in SCI

In the sham group (n = 20), mice received bilateral laminectomy without damage to the spinal cord and treated with saline injection in the wound. Besides, SCI mice were randomly divided into four groups, and 20 mice in each group were respectively treated with phosphate buffer saline (SCI group), or 20 μg hucMSC-EVs (SCI + hucMSC-EVs group), or 20 μg CD73 +hucMSC-EVs (SCI + CD73 +hucMSC-EVs group), or 20 ng rmCD73 (recombinant mouse CD73, Beijing Baiao Lai Bo Technology Co., Ltd., Beijing, China). For topical administration, 20 μg EVs were diluted to 50 μg/ml by phosphate buffer saline in a 1 ml syringe, which was injected into the wound at the spinal cord injury site of T9 exposed spinal cord once a day and for 10 days post injury [31].

### In vivo biodistribution study

To test the biodistribution of EVs, images were taken with an in vivo imaging system (IVIS) (IVIS Spectrum, PerkinElmer, USA) at 24 h post-injection and the fluorescence intensities were analyzed by Living Image Software. Briefly, 6 nude mice were purchased from SLAC Company (Shanghai, China). CD73+hucMSC-EVs were labeled with DiR for further administration. Three mice were treated with intraperitoneal injection, and 3 mice were injected in situ around the injured spinal cord. After 24 h, nude mice were anesthetized, and were analyzed by bioluminescence imaging in vivo. Anteroposterior and lateral graphs were taken to determine locations of labeled EVs.

### Behavioral tests

Basso Mouse Scale (BMS) open-field score and Basso-Beattie-Bresnahan (BBB) score were used to evaluate the functional recovery of hind limb locomotion on 1, 3, 7, 14, 21, 28, 35 and 42 days post injury by two well-trained observers who were blinded to the experimental animal grouping. For these tests, mice were allowed to move freely in an open field for 4 min, and average locomotor scores were calculated and recorded [32, 33].

### Measurement of ATP in cerebrospinal fluid (CSF)

Mice were anaesthetized by isoflurane and oxygen, and performed by direct cisterna magna puncture with a glass capillary tube as described previously [34, 35]. And then, CSF was collected for ATP measurement using the ATP Assay Kit (Beyotime, Shanghai, China).

### Immunohistochemistry staining

On day 42, animals were euthanized and were perfused with 4% paraformaldehyde through the left ventricle as previously described [36, 37].

The whole spinal cords were rapidly removed and embedded in paraffin. Longitudinal sections were produced at 5 μm from a 1 cm length of spinal cord centered on the injury epicenter. Slides of each spinal cord were stained with Haematoxylin and Eosin (H&E), Nissl staining, and TdT-mediated DUTP nick end labeling (TUNEL) staining.

### Flow cytometry, cytokine analysis, and ELISA assay

To isolate microglia, spinal cord tissues were removed and minced with scissors in ice‐cold Dulbecco's Modified Eagle Medium (DMEM) (Invitrogen). Thereafter, a monocular suspension was formed by digesting with 0.25% trypsin (Invitrogen) in a water bath at 37 °C for 30 min, and then were co-stained for CD206 (an M2 microglia biomarker) and CD86 (an M1 microglia biomarker) for 45 min at room temperature following the manufacturer’s instructions [38]. The samples were detected using FACSAria III flow cytometer (BD Biosciences, San Jose, CA, USA) and then analyzed by FlowJo software v.7.6.1 (https://flowjo.com/).

Furthermore, the Bio-Plex system (Bio- Rad, CA) with a 23-plex cytokine array kit (Bio-Rad) was used for cytokine analysis of spinal cord samples.

Similarly, the levels of cytokines were also measured by ELISA in spinal cord samples, including IL-1β (cat. no. P1303), IL-6 (cat. no.P1328), TNF-α (cat. no. PT516), and IL-10 (cat. no. PI525), using protocols supplied by the manufacturer (Beyotime Biotech., Jiangsu, China).

## Results

### Isolation and characterization of hucMSC-EVs and CD73+hucMSC-EVs

**Fig. 1:**
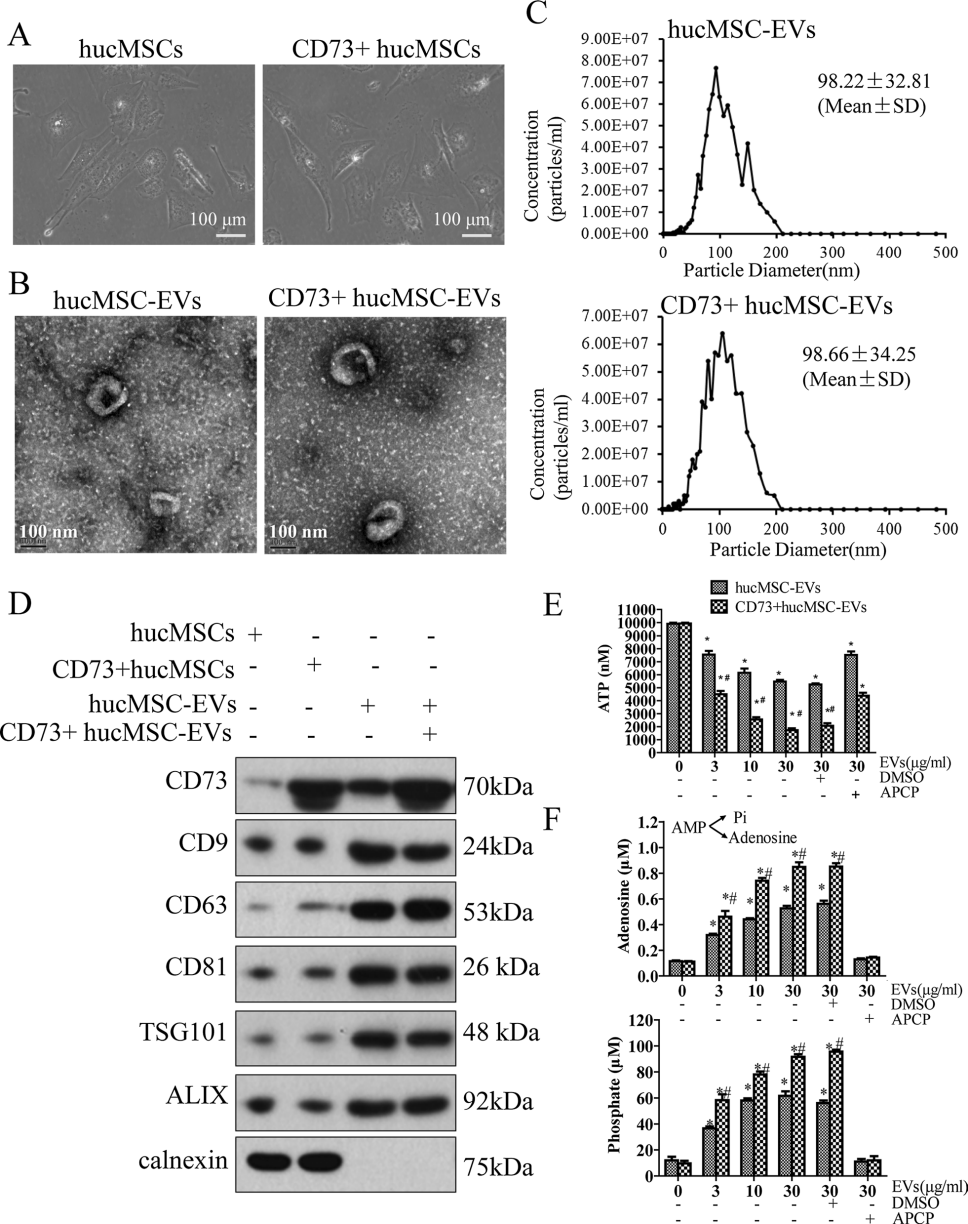
Characteristics of hucMSC-EVs and CD73 +hucMSC-EVs. **A** Representative images of hucMSCs and CD73+ hucMSCs are observed under microscopy. **B** Representative images of hucMSC-EVs and CD73+ hucMSC-EVs are observed under transmission electron microscopy (TEM). **C** Size distribution of extracellular vesicle is measured by nanoparticle tracking analysis (NTA, ZetaView, Particle Metrix Inc., German). **D** Western-blotting analysis of indicated proteins is detected, including the modified protein of CD73, exosomal positive biomarkers of CD9, CD63, CD81, TSG101, and ALIX, and exosomal negative biomarker of calnexin. (E and F) The ATP and AMP hydrolytic activities of the indicated dose of hucMSC-EVs or CD73+hucMSC-EVs with or without 100 μM APCP are measured in vitro (n = 3)

Under light microscope, hucMSCs derived from umbilical cord tissue and the modified CD73+hucMSCs by lentiviral transduction appeared similar growth characteristics (Fig. 1A). The hucMSC-EVs and CD73+hucMSC-EVs displayed classic type of spherical morphology under the transmission electron microscopy (Fig. 1B). NTA showed that the diameters of hucMSC-EVs and CD73+hucMCS-EVs were 103.41 ± 42.03 and 106.51 ± 53.99 nm, respectively (Fig. 1C). The protein concentration in the EVs was 2.5 μg/μl detected by BCA kit. In addition, western blot revealed that the surface markers (CD9, CD63, CD81, TSG101, and ALIX) were all positive in hucMSC-EVs and CD73 +hucMSC-EVs, but calnexin was negative in hucMSC-EVs and CD73+hucMSC-EVs. They are acknowledged biomarkers of testing EVs. Moreover, the expression of CD73 was significantly higher in CD73+hucMSCs and CD73+hucMCS-EVs (Fig. 1D).

### Effects of CD73+hucMCS-EVs on hydrolyzing ATP into adenosine and promoting microglia cell polarization from M1 to M2 phenotype in vitro

In the detection of the ATP and AMP hydrolytic activities, it showed that hucMCS-EVs and CD73+hucMCS-EVs could dose-dependently hydrolyze ATP and AMP, which was significantly mitigated by the CD73-specific inhibitor APCP (Fig. 1E and F). Moreover, hydrolytic effects of CD73+ hucMCS-EVs performed significantly stronger than that of hucMCS-EVs.

**Fig. 2:**
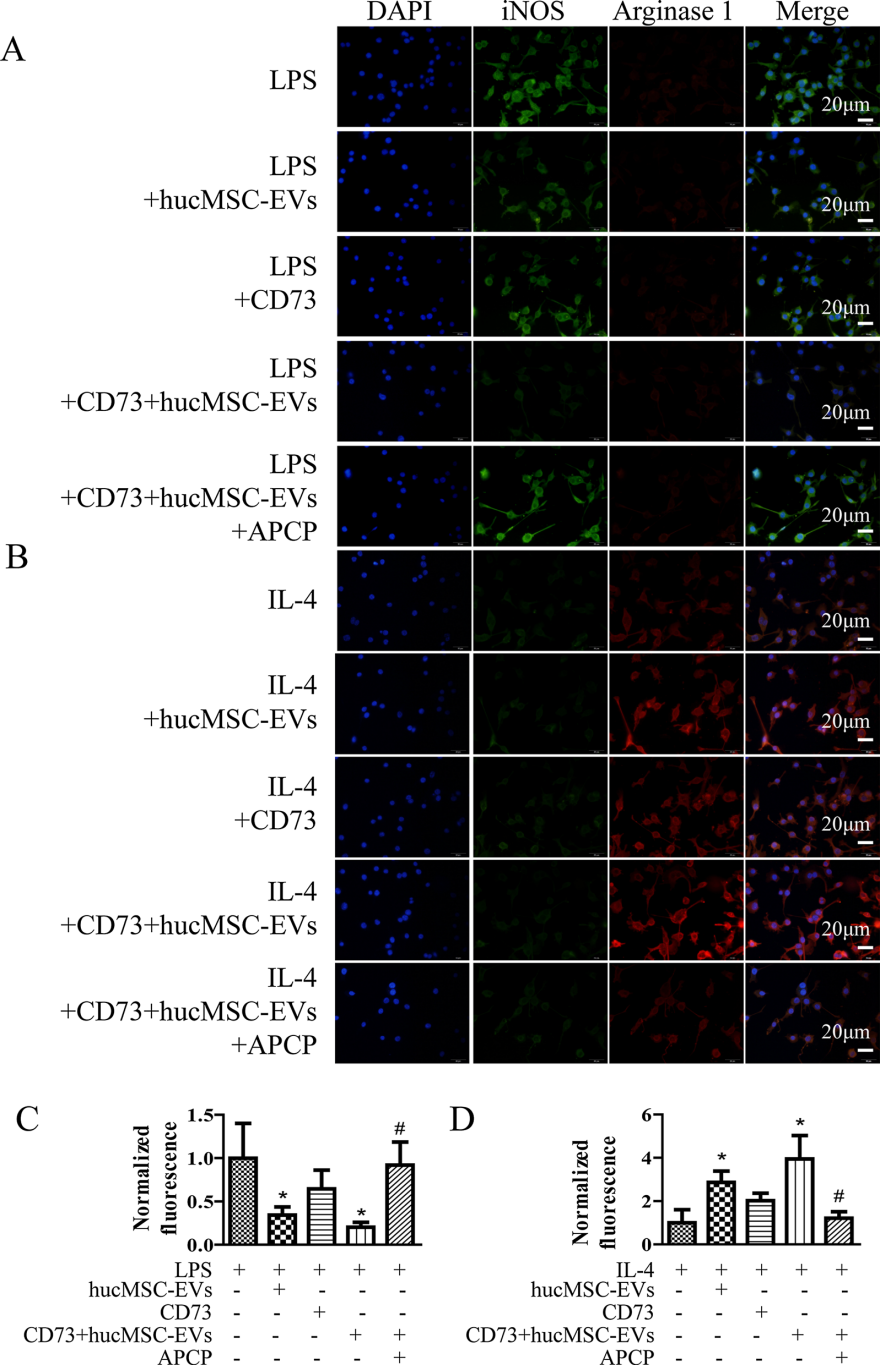
CD73+ ucMSC-EVs promote M2 polarization and inhibit M1 polarization in vitro. **A** The effects of 30 ng/ml CD73, 30 μg/ml hucMSC-EVs and 30 μg/ml CD73+hucMSC-EVs with or without 100 μM APCP on the change of M1 subsets in BV2 cells treated with 1 μg/ml LPS are determined by immunofluorescences. **B** The effects of 30 ng/ml CD73, 30 μg/ml hucMSC-EVs and 30 μg/ml CD73+ hucMSC-EVs with or without 100 μM APCP on the change of M2 subsets in BV2 cells treated with 5 μg/ml IL-4 are determined by immunofluorescences. **C** and **D** Fluorescent intensities are normalized to fluorescent levels in LPS group or IL-4 group (*p < 0.05 versus LPS/IL-4, #p < 0.05 versus LPS/IL-4 + CD73+hucMSC-EVs, n = 3)

To reduce the release of neuroinflammatory factors, the M1/M2 balance of microglia has far-reaching significance. In this study, BV2 cells from mice were isolated and cultured for 7 days, and then were induced with 1 μg/ml LPS or 5 μg/ml IL-4 for the polarization to M1 or M2 cells, respectively. And then, iNOS and Arginase 1 were selected as the surface marker of M1/M2 cells (Fig. 2). Finally, it showed that hucMSC-EVs, CD73+hucMSC-EVs, and CD73 could reduce the expression of iNOS induced by LPS. CD73+hucMSC-EVs decreased it the most, while its effect could inhibited by APCP. On the other hand, it also showed that CD73+hucMSC-EVs significantly increased the expression of Arginase 1, which was reversed by APCP.

### CD73+hucMSC-EVs augmented M2 polarization and mitigate M1 polarization via A_2b_ adenosine receptor activation

**Fig. 3:**
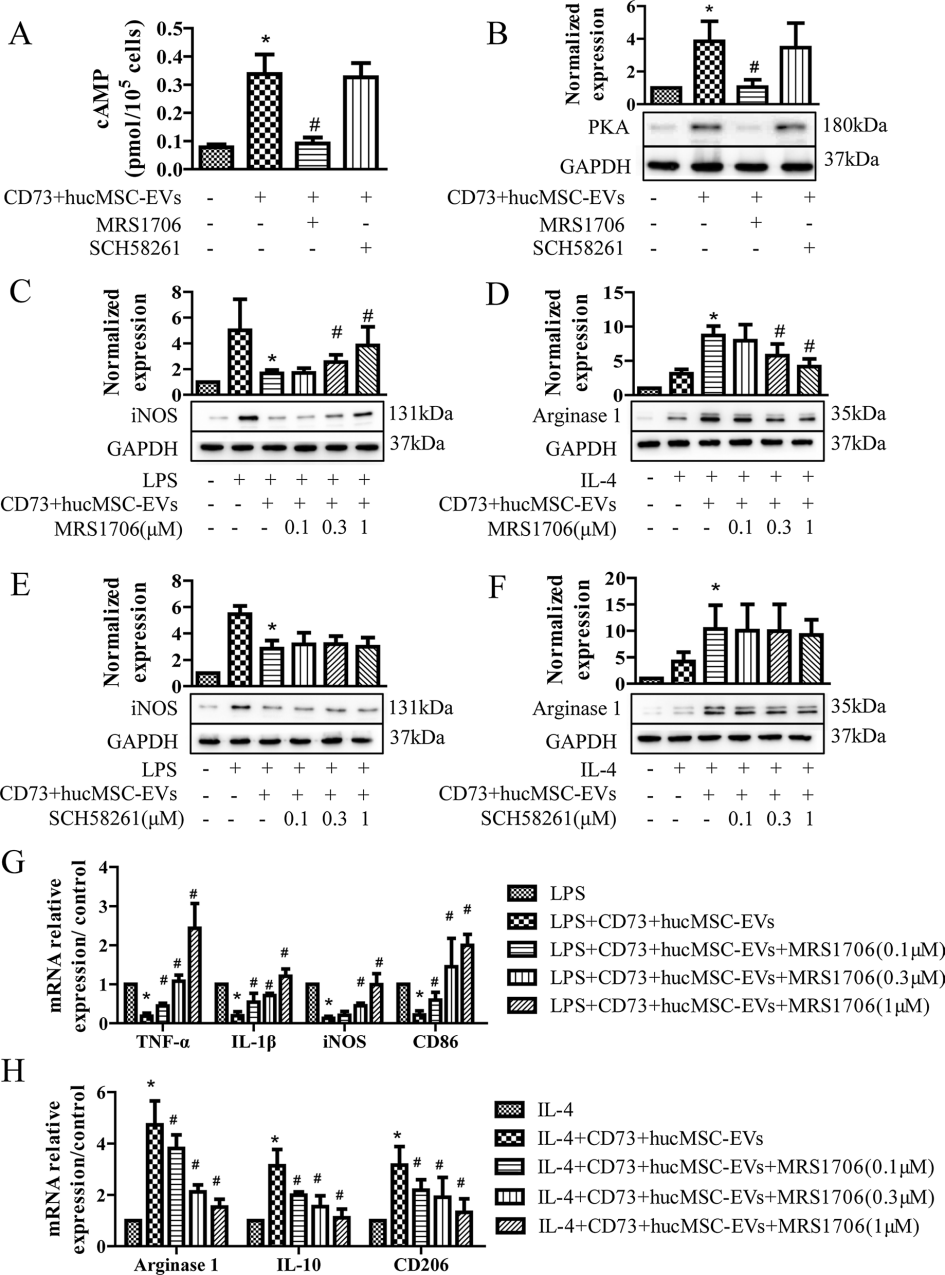
CD73+ ucMSC-EVs augment M2/M1 polarization via A_2b_ adenosine receptor activation. **A** and **B** BV2 cells are treated with 1 μg/ml LPS in the presence or absence of 30 μg/ml CD73+hucMSC-EVs, and together with 1 μM MRS1706 (A_2b_R inhibitor) or 1 μM SCH58261 (A_2a_R inhibitor). **A** Intracellular cAMP levels are measured after 10 min stimulation (n = 5). **B** PKA protein expression in BV2 cells is detected (n = 3). (*p < 0.05 versus control, *#p* < 0.05 versus CD73+ hucMSC-EVs) **C**–**F** BV2 cells are treated with 1 μg/ml LPS or 5 μg/ml IL-4 in the presence or absence of 30 μg/ml CD73+hucMSC-EVs, and together with the indicated dose of MRS1706 or SCH58261. The iNOS/Arg-1 protein expression in BV2 cells was either investigated. **G** The mRNA relative expression level of M1 phase is detected, including TNF-α, IL-1β, iNOS, and CD86. **H** The mRNA relative expression level of M2 phase is detected, including arginase 1, IL-10, and CD206. (*p < 0.05 versus LPS/IL-4, *#p* < 0.05 versus LPS/IL-4 + CD73+hucMSC-EVs, n = 3)

**Fig. 4:**
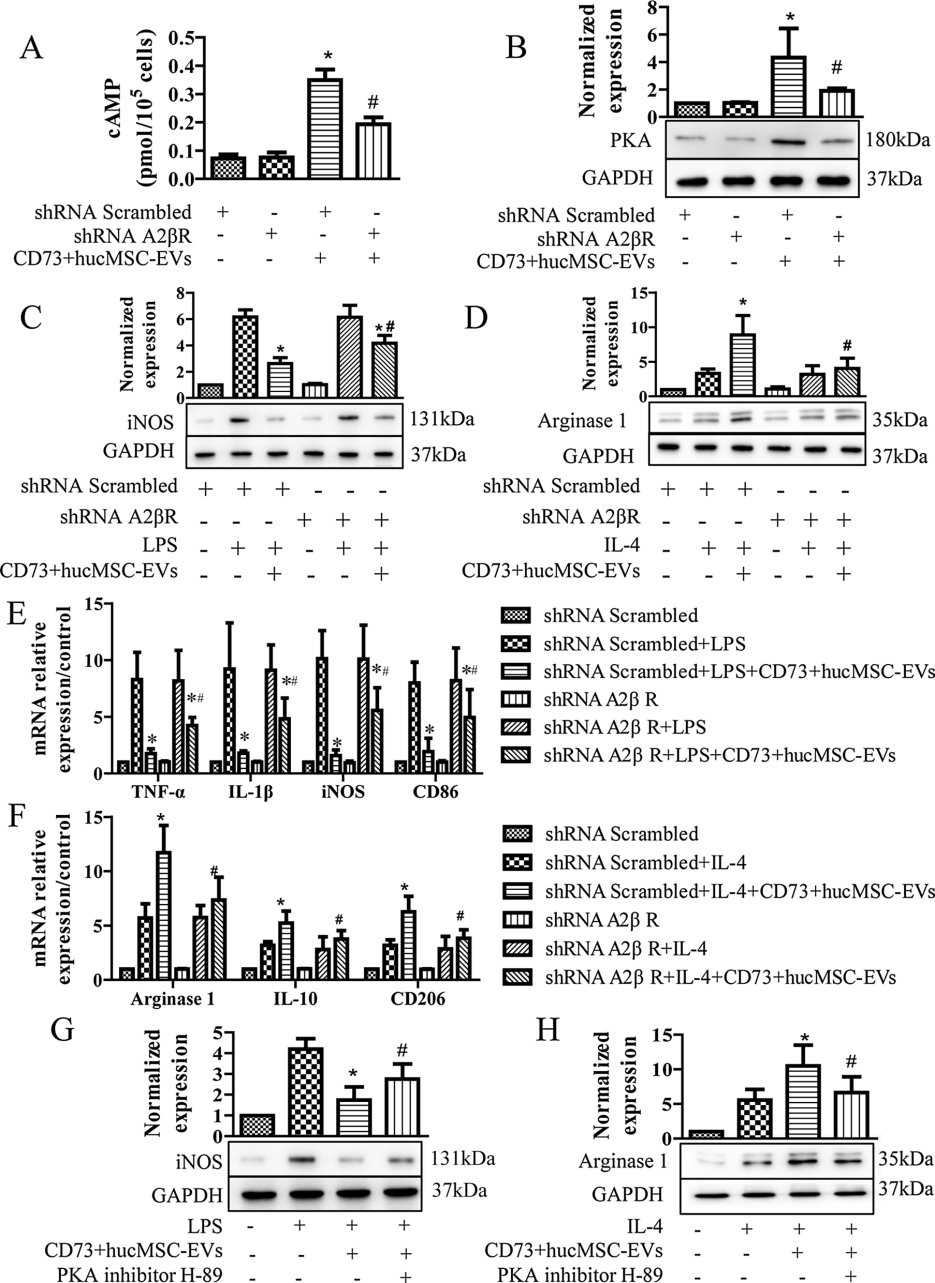
A_2b_R knockdown cells and PKA inhibitor reverse the stimulatory effect of CD73+hucMSC-EVs. **A**–**F** BV2 cells are permanently transfected with scrambled shRNA or A_2b_R shRNA. Cells are then treated with 1 μg/ml LPS or 5 μg/ml IL-4 in the presence or absence of 30 μg/ml CD73+ hucMSC-EVs. **A** and **B** Intracellular cAMP levels are measured after 10 min stimulation (n = 5). PKA protein expression is detected (n = 3). **C** and **D** The iNOS/Arg-1 protein expression in BV2 cells is investigated. **E** and **F** The mRNA relative expression level of M1/M2 phase is either detected. (*p < 0.05 versus shRNA scrambled/A_2b_R + LPS, *#p* < 0.05 versus shRNA scrambled + CD73 + hucMSC-EVs). **G** and **H** The iNOS/Arg-1 protein expression in BV2 cells is investigated in presence or absence of 10 μM H-89 (PKA inhibitor)

To verify the downstream target of overexpression of CD73, we used SCH58261 and MRS1706 as inhibitors of A_2a_ and A_2b_ receptors, respectively. A_2_Rs are known to mediate inflammation in microglia, and A_2_Rs are Gs-linked protein and increase cAMP. In Fig. 3A, 30 μg/mL CD73+hucMCS-EVs significantly increased the level of cAMP. And it was significantly reduced by 1 μM MRS1706 (A_2b_R inhibitor) but not 1 μM SCH58261 (A_2a_R inhibitor). For downstream targets of cAMP, PKA also increased after treating with CD73+hucMCS-EVs, and it was significantly prevented by MRS1706 but not SCH58261 (Fig. 3B). In addition, Arginase 1 and iNOS were often used as markers of microglia subtypes, since arginase 1 could effectively downregulate nitric oxide production caused by iNOS and activate anti-inflammatory function. When treated with LPS, CD73+hucMCS-EVs significantly decreased the level of iNOS; and when treated with IL-4, CD73+hucMCS-EVs significantly increased the level of Arginase 1. Interestingly, MRS1706 performed a dose dependent way on inhibiting the effect of CD73+hucMCS-EVs (Fig. 4C, D). In contrast, SCH58261 showed little effect on CD73+hucMCS-EVs (Fig. 4E, F). Furthermore, the expression of M1 mRNA markers (TNF-α, IL-1β, iNOS, and CD86), and the expression of M2 mRNA markers (Arginase, IL-10, and CD206) were detected. It also showed similar outcomes to the western blot results (Fig. 4G, H). As a result, CD73 + hucMCS-EVs might enhance M2 polarization and moderate M1 polarization via A_2b_ adenosine receptor activation, but not A_2a_ adenosine receptor.

### A_2b_R knockdown cells and PKA inhibitor reversed the stimulatory effect of CD73+hucMSC-EVs on alternatively activated microglia

A_2_bR was knockdown by permanently transfected A_2b_R shRNA. Treated with LPS and CD73+hucMCS-EVs, the concentration of cAMP in shRNA A_2b_R group was significantly decreased when compared with shRNA scrambled group (Fig. 4A). The standardized expression level of PKA was also significantly weakened in A_2b_R shRNA group when treated with CD73+hucMCS-EVs (Fig. 4B). In addition for M1/M2 subtypes, CD73+hucMCS-EVs downregulated the level of iNOS induced by LPS in both shRNA scrambled group and shRNA A_2b_R group, but the level of iNOS in shRNA A_2b_R group was significant higher than that in shRNA scrambled group. And the level of Arginase 1 was significantly lower in shRNA A_2b_R group than that in shRNA scrambled group when treated with CD73+hucMCS-EVs and IL-4 (Fig. 4C and D). Similarly, the mRNAs of M1/M2 markers was observed in A_2b_R knockdown cells, and the results were concurred with the western blot findings (Fig. 4E and F). As a result, it confirmed that A_2b_R was important in the effects of CD73+hucMCS-EVs.

Meanwhile, cAMP signals proceed via intracellular activation of PKA, and the PKA inhibitor H-89 is used to determine this downstream target. It showed that the level of iNOS was increased after LPS induction. And CD73+hucMCS-EVs decreased the iNOS expression, which was inhibited by H-89. It also showed that H-89 decreased the upregulating effect of CD73+hucMCS-EVs on the level of Arginase 1 (Fig. 4G). Thus, A_2b_R was identified as the receptor of adenosine produced by CD73+hucMCS-EVs, and downstream biomarkers might be activated via A_2b_R/cAMP/PKA signaling pathway.

### CD73+hucMSC-EVs ameliorated SCI and decreased intracellular cAMP level in mice

**Fig. 5:**
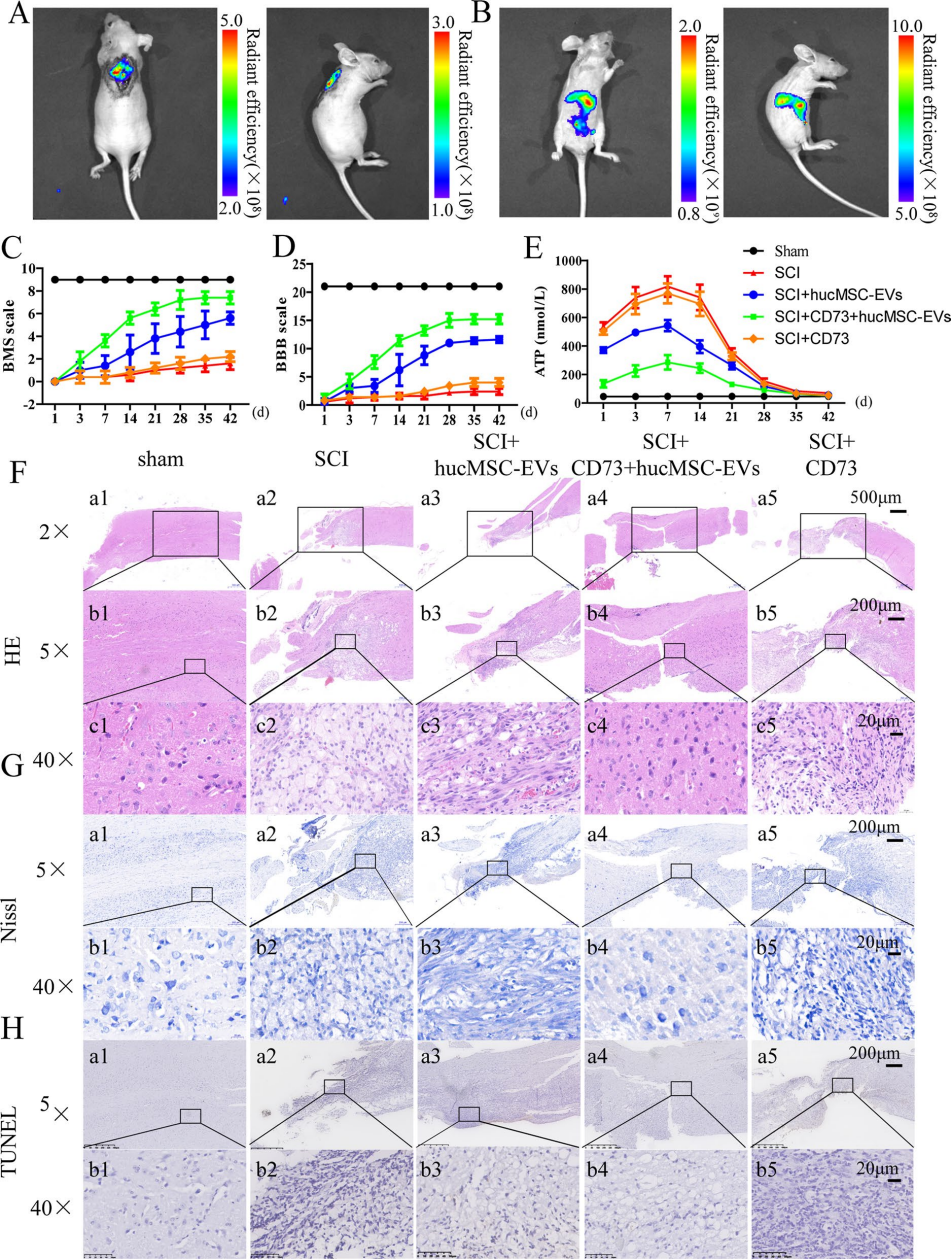
CD73+hucMSC-EVs ameliorate SCI and decrease intracellular cAMP levels in mice. (A and B) In vivo biodistribution of DiR-labeled CD73+hucMSC-EVs. Mice are analyzed at 24 h after **A** subcutaneous injection of EVs around injured spinal cord and **B** intraperitoneal injection of EVs. **C** BMS scores at different time-point after spinal cord injury. **D** BBB scores at different time-point after spinal cord injury. **E** cAMP levels from cerebrospinal fluid at different time-point after spinal cord injury. **F** Histological images (H&E staing), **G** Nissl staining and (H) TUNEL staining of longitudinal sections of injured spinal cords on day 21

Prior to exploring the effects of EVs, in vivo biodistribution of EVs was investigated by IVIS imaging analysis. The fluorescence signal of DiR-labeled EVs remained well around the wound 24 h after subcutaneous injection (Fig. 5A). However, 24 h after intraperitoneal injection, it presented intense signals in the abdominal cavity and mainly in liver and kidneys, but it showed almost none fluorescence signals around the spinal cord. (Fig. 5B). Therefore, EVs were directly injected into the wound in the following study.

We established a mouse spinal cord injury model according to Allen's method. Saline, hucMSC-EVs, CD73+hucMSC-EVs, or recombinant mouse CD73 was respectively treated for 10 days after the injury. Firstly, we recorded the body weight of each mouse. There was a significant decrease in the body weight of all groups on day 3 after the injury, and mice in SCI + CD73+hucMCS-EVs group gradually recovered with a significant increase in body weight, when compared with other groups suffered from SCI (Additional file 2: Fig. S1A). And then, to compare the different levels of motor function of the five groups of mice, we recorded BMS scores and BBB scores at different times at 1, 3, 7, 14, 21, 28, 35 and 42 days after operation. The exercise scores were calculated according to the scoring criteria, and Excel was used to record and plot the plot lines of different groups with BMS scores and BBB scores. It showed that the SCI + CD73+hucMCS-EVs group had a significant improvement on post-SCI motor dysfunction than SCI group. The SCI + CD73+hucMCS-EVs group also showed a better result than the SCI + hucMCS-EVs group and the SCI + CD73 group (Fig. 5C, D). In addition, cerebrospinal fluid of mice was extracted to detect ATP concentration under pathological conditions on the corresponding 1, 3, 7, 14, 21, 28, 35 and 42 days. The concentration of sham group was basically unchanged. ATP level in SCI + CD73+hucMCS-EVs group was significantly increased than in SCI group. And ATP levels in SCI + +hucMCS-EVs group were moderately increased. Interestingly, the ATP level upgraded within 1–7 days, and slowed down after day 14 due to the withdrawn of treatment on the day 10 (Fig. 5E).

Furthermore, we selected the spinal cord tissue of the mouse model segment for HE staining, Nissl staining, and TUNEL staining on day 42 post injury. The SCI group presented that the Nissl body neuron revealed to be disordered, swelling, and vacuolar structure. HucMCS-EVs reduced the injury, and CD73+hucMCS-EVs performed better (Fig. 5F, G). In addition, TUNEL-positive cells were obviously reduced in SCI + CD73+hucMCS-EVs group, when compared to other groups (Fig. 5H).

### CD73+hucMSC-EVs regulated M1/M2 polarization and pro-inflammatory cytokines after SCI in mice

**Fig. 6:**
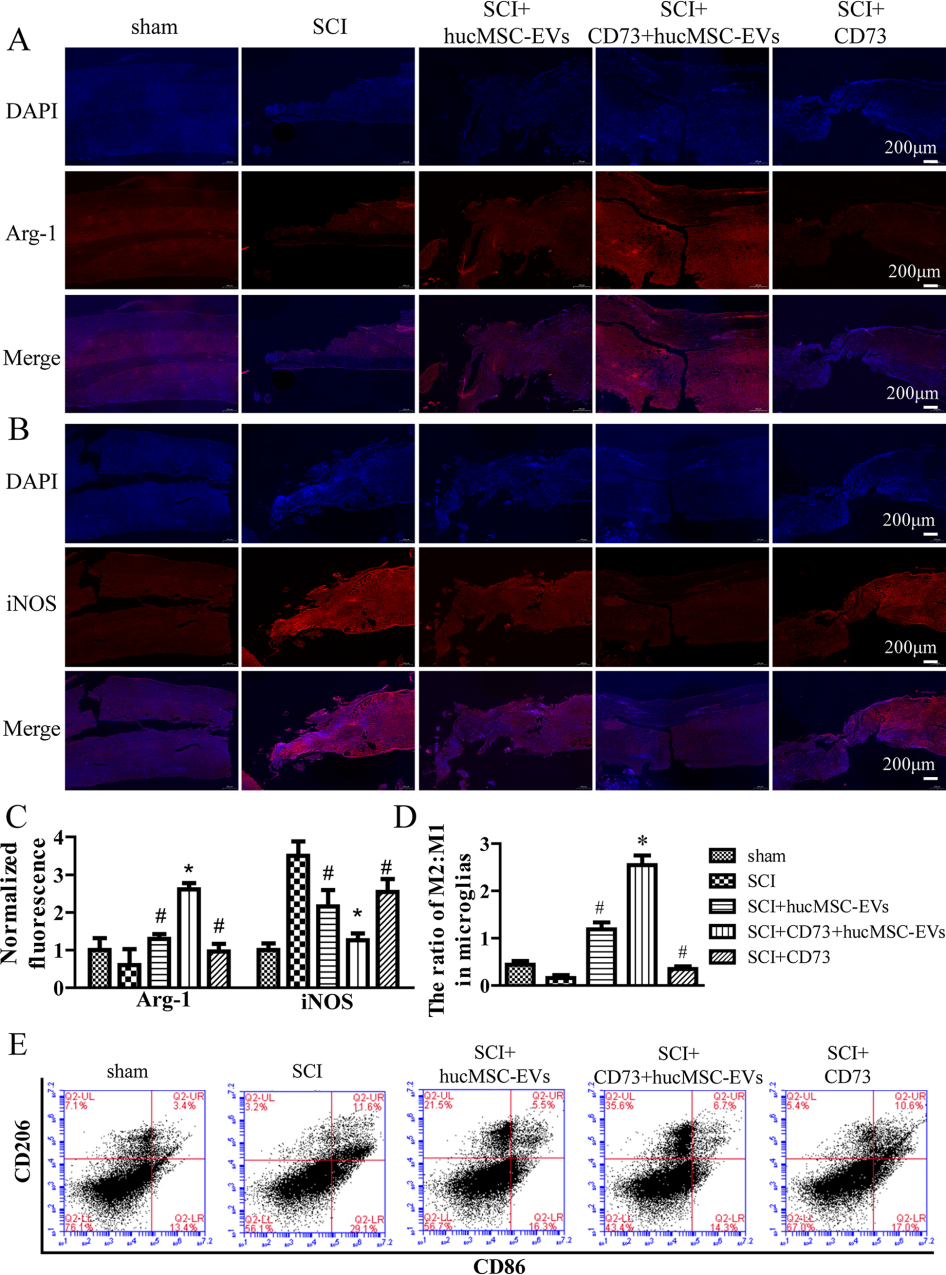
CD73+hucMSC-EVs regulate M1/M2 polarization of microglia in mice. **A** and **B** Changes of arginase-1 and iNOS are determined by immunofluorescence in different groups at × 5 magnification. **C** Fluorescent intensities are normalized to the sham group. (*p < 0.05 versus SCI group, #p < 0.05 versus SCI + CD73+hucMSC-EVs group, n = 5). **D** and **E** Representative dot spot of flow cytometry for microglia/macrophage subsets is shown. CD206 and CD86 are selected as biomarkers of M2 and M1 microglia, respectively. The data are calculated as M2:M1. (*p < 0.05 versus SCI group, #p < 0.05 versus SCI + CD73+hucMSC-EVs group, n = 5)

To determine the ratio of M2 and M1 microglia in spinal cord samples of mice, immunofluorescence was used for mice executed on day 7 after SCI. Antibodies of Arg-1 and iNOS were applied for immunofluorescence in accordance with antibodies used in vitro. CD73+hucMCS-EVs significantly reduced iNOS positive cells and improved Arg-1 positive cells in vivo (Fig. 6A, B, and Additional fiile 2: Fig. S1B). In addition, flow cytometry was also employed to test CD206 and CD86, biomarkers of M2 and M1 microglia, respectively. And it showed that the calculated M2:M1 was significantly increased in SCI + CD73+hucMCS-EVs group than other groups (Fig. 6D, E).

**Fig. 7:**
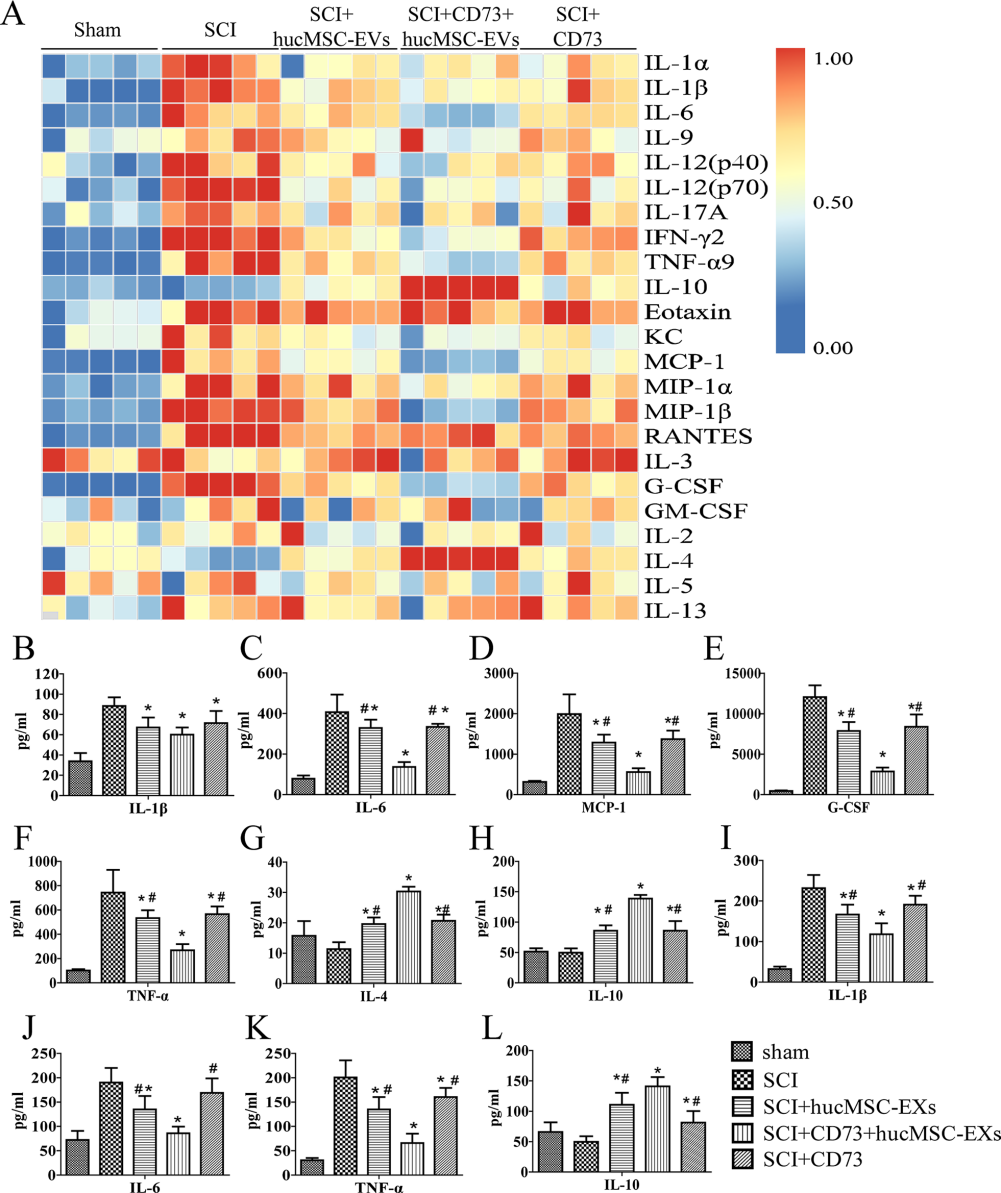
CD73+hucMSC-EVs regulate the production of proinflammatory cytokines after SCI. **A** Cytokines of spinal samples from SCI mice are quantitative analyzed by Bio-Plex system 3d after SCI and hucMSC-EVs/CD73+hucMSC-EVs treatment. **B**–**H** The protein levels analyzed by Bio-Plex system, such as IL-1β, IL-6, TNF-α, IFN-γ, MCP-1 and MIP-1β are significantly decreased, while IL-4 and IL-10 are significantly increased. **I**–**L** The protein levels, including IL-1β, IL-6, TNF-α, and IL-10, are also analyzed by ELISA. (*p < 0.05 versus SCI group, #p < 0.05 versus SCI+CD73+hucMSC-EVs group, n = 5)

Additionally, 7 days after treatments, cytokines in mice spine samples were quantitatively analyzed with bio-plex system. After the cytokine content of each group was standardized, a heatmap was established for each group (Fig. 7A). Pro-inflammatory cytokines, such as IL-1β, IL-6, TNF-α, MCP-1, IFN-α, and MIP-1β, were significantly reduced in SCI+CD73+hucMCS-EVs group. Anti-inflammatory cytokines, such as IL-4 and IL-10, were significantly increased in SCI+CD73+hucMCS-EVs group (Fig. 7B–H). Similarly, the releases of IL-6, IL-1β, TNF-α, and IL-10 in spinal cord tissues of SCI mice were also detected by ELISA. Compared with the SCI group, the levels of TNF-α, IL-6, and IL-1β were increased in SCI+CD73+hucMCS-EVs group (*p* < 0.05), and the important anti-inflammatory cytokine, IL-10 (*p* < 0.05) was markedly decreased (Fig. 7I–L). Collectively, CD73+hucMCS-EVs upregulated the polarization of M2:M1 and alleviated inflammatory response in the repair of spinal cord injury.

### CD73+hucMSC-EVs reduced activation of astrocytes mainly expressed as the A1 phenotype after SCI in mice

After SCI, microglia might release molecules that trigger astrocyte proliferation and astrocyte scar formation [39]. To determine the activation astrocytes, we tested GFAP, which represents the astroglial scar and is expressed throughout the entire injury site. It showed a significant expression of GFAP-positive cells in SCI group, and GFAP-positive cells were significantly decreased in CD73+hucMCS-EVs group when compared with other mice after SCI. Furthermore, to examine the subtypes of astrocytes, which are defined as A1 phenotype (inflammatory astrocyte) and A2 phenotype (tissue repair astrocyte), C3 and S100A10 were used as biomarkers, respectively [40]. As a result, C3-positive cells were significantly increased in SCI group but significantly inhibited in CD73+hucMCS-EVs group (Additional file 3: Fig. 2A). However, there was no significant difference in S100A10-positive cells among groups (Additional file 3: Fig. 2B).

## Discussion

**Fig. 8:**
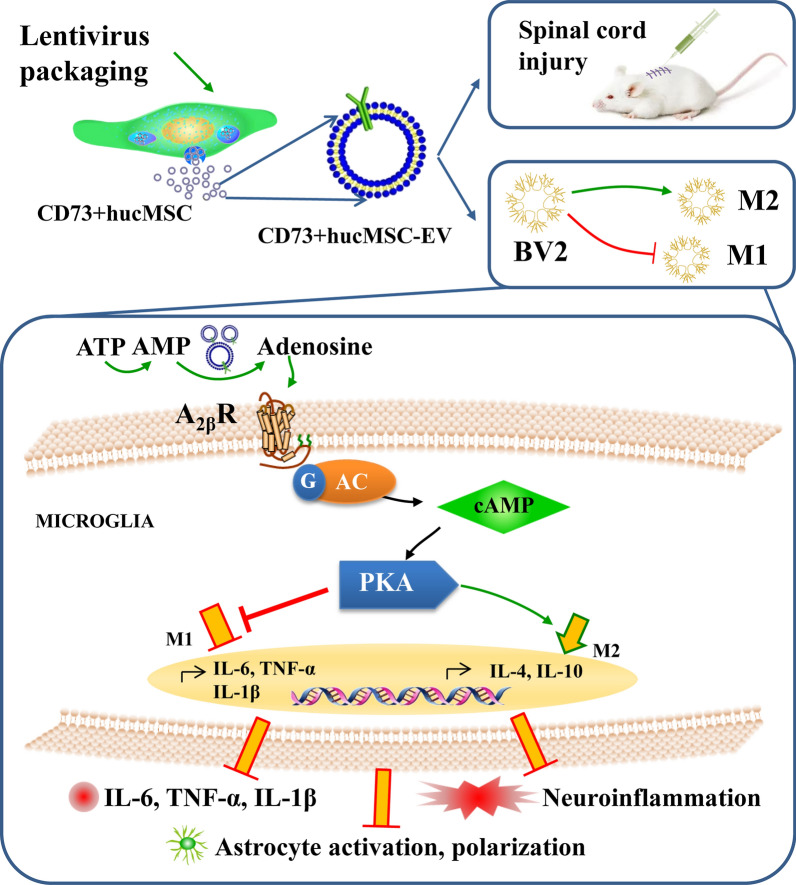
Schematic diagram showing the effects of CD73+hucMSC-EVs on microglia

In this study, we established CD73 overexpressed engineered EVs as the Nano drug carriers, and reported that CD73+hucMSC-EVs treatment alleviated inflammation after spinal cord injury in mice and regulated macrophages/microglia M2:M1 polarization in vitro. CD73+hucMSC-EVs reduced concentration of ATP and promoted level of adenosine, which further activated A2bR and cAMP/PKA signaling pathway. Therefore, our results provide evidences that engineered EVs as CD73+hucMSC-EVs could protect against inflammation in SCI (Fig. 8).

It is well known that inflammation plays an important role in the pathogenesis of SCI [41, 42]. Inflammation and tissue damage can generate excessive systemic ATP into the extracellular space, and trigger a cascade of secondary injury [43, 44]. Extracellular ATP can be degraded to immunosuppressive adenosine by two steps by membrane-bound enzymes of CD39 and CD73 [45], which is pivotal in acute inflammation [46]. And CD73 is the rate-limiting enzyme in the final step of the conversion of AMP to adenosine [47]. Recently, it is reported that an overexpression of CD73 in BV2 cells imparts neuroprotective effects by mediating macrophages/microglia polarization [26]. However, whether this enzyme can be applied as a drug for SCI in vivo via regulating the levels of adenosine has never been investigated. Of note, CD73 is reported as an emerging immune checkpoint and an ideal target for cancer treatment [12, 13]. In other words, an overexpression of CD73 might cause progression and recurrence of carcinoma [48]. As a result, the strong immunosuppression of CD73 might limit its application as a systemic medication, but a local delivery of CD73 with nanocarriers might be an alternative approach.

EVs can be utilized to transport genetic material or drugs to target cells. EVs are nanometer-sized, lipid-bilayer-enclosed vesicles, which make them able to permeate the BBB [49]. It has been developed for the treatment of various diseases including cancer and CNS disorders [50, 51]. In addition, the MSC-derived EVs (MSC-EVs) possess the original characteristics from MSCs, such as regenerative function and damage reduction effects. Compared to MSCs, MSC-EVs have different advantages including greater stability and handling, a lower chance of immunological rejection, and no risk of malignant transformation [52]. It has been shown MSC-EVs play a critical role in repairing SCI through promoting angiogenesis and axonal growth, and regulating inflammation and the immune response [31]. Consequently, hucMSC-EVs were chosen as the nanocarriers for CD73 in this study, and CD73+hucMSC-EVs would not only have the strength of hucMSC-EVs, but also possessed the anti-inflammatory ability of CD73.

To prepare the engineered EVs, lentiviral transduction is used to harvest CD73+hucMSCs, and then CD73+hucMSC-EVs are isolated as nanodrugs. It showed that CD73+hucMSC-EVs dose-dependently hydrolyze ATP and AMP, and the produced adenosine was bonded to A_2b_R. Adenosine receptors are classified to A_1_R, A_2a_R, A_2b_R and A_3_R, which produce different physiological effects through biological signal transduction. A1R and A3R are coupled to Gi, which inhibits the level of cAMP, but A2aR and A2bR are coupled to the stimulatory G alpha protein (Gs), increasing the levels of cAMP [53]. Moreover, the A_2b_R activation on macrophages is critically required for the stimulatory effect of adenosine on IL-10 production and suppression of nitric oxide release [54, 55]. And studies show that the A_2b_R activation can increase alternative M2/M1 polarization by adenosine stimulation [56, 57]. Therefore, we used selective antagonists of A_2a_R and A_2b_R, and A_2b_R knockdown cells, and therefore demonstrated that the A_2b_R was responsible for microglia alternative activation stimulated by adenosine, which is hydrolyzed by CD73+hucMSC-EVs.

After A_2b_R activation, the level of cAMP is elevated. And protein kinase A (PKA) is an intracellular receptor of cAMP. In this study, we showed A_2b_R signaling increased the expression of cAMP/PKA by three methods, including A_2b_R antagonist treatment, A_2b_R knockdown cells, and PKA inhibitor treatment. Other studies also showed that A2bR activation induces cAMP/PKA signaling pathway [58–60]. Therefore, the activated PKA suppressed the activation of M1 and increased the polarization of M2, which also has been reported [61–63]. Thus, the cascaded pro-inflammatory cytokines were downregulated, such as TNF-α, IL-1β, and IL-6; while the anti-inflammatory cytokines are upregulated, such as IL-10 and IL-4 [64, 65].

Subsequently, inflammatory microenvironment coupled with the cascade effect of M1 microglia contributed to the development of the secondary injury phase. It is known that activated microglia can induce the transformation of astrocytes into the A1 phenotype by releasing cytokines, such as interleukin-1 alpha (IL-1α), tumor necrosis factor (TNF) [66]. Generally after SCI, there were mainly of the A1 phenotype and less of the A2 phenotype, and A2 astrocytes are induced by damaged neurons via the secretion of prokineticin-2 [67]. In this study, the A1 phenotype astrocytes were significantly expressed in SCI group but less in CD73+hucMCS-EVs treated group, and A2 phenotype astrocytes showed no significant difference among groups after SCI. It can be explained that CD73+hucMCS-EVs hydrolyzed ATP and AMP into adenosine, which inhibited the inflammatory microenvironment. Since microglia are the primary immune sentinels in central nervous system [68], the activation of spinal microglia was reported the most significant at day 7 after injury, while the activation of spinal astrocytes in rats was most significant at day 14 after injury [69]. Therefore, the adenosine produced by CD73+hucMCS-EVs might firstly affected with microglia, which significantly reverted the ratio of M1/M2 reactive microglia, rather than A1/A2 astrocyte transformation.

However, this study has several limitations. On one hand, hucMSC-EVs contain a large component of protein, mRNA, and miRNA, which also can show function of modulation of macrophage phenotype, and suppression of inflammatory action [70]. Thus, it is unclear the importance of the contribution of the components of hucMSC-EVs rather than that of CD73 overexpression. This study compared the engineered CD73+hucMSC-EVs to hucMSC-EVs, so as to reduce the differences. On the other hand, the tumorigenicity of CD73 after a long term usage in vivo is still unknown, so side effects of CD73 as a drug need further study. With EVs as nano drug carriers, we believe side effects could be reduced since EVs can be designed for targeting cells by expression of specific proteins on the surface. Lastly, CD73+hucMSC-EVs were injected into the wound in this study, which was an invasive treatment. Alternatively, it could be resolved by constructing collagen scaffold that contains and delivers CD73+hucMSC-EVs, which can be implanted on the surface of spinal cord during the decompression surgery for one-step repair.

## Conclusions

Our research firstly establishes engineered EVs as the Nano drug carriers overexpressing CD73 that facilitates the hydroxylation of ATP to adenosine. It is demonstrated CD73+hucMSC-EVs might ameliorate inflammation after spinal cord injury and regulating macrophages/microglia M2:M1, by activating A_2b_R and cAMP/PKA signaling pathway.

## Supplementary Information

**Additional file 1: Table S1**. Sequence of the real-time PCR primers.

**Additional file 2: Figure S1**. Body weights of mice and Immunofluorescence of Arginase-1 and iNOS. (A) Body weights of mice. There was a significant decrease in the body weight of all groups on day 3 after the injury. And then, mice in SCI+CD73+hucMCS-EVs group gradually recovered with a significant increase in body weight, when compared with other groups suffered from SCI. (B) Changes of arginase-1 and iNOS are determined by immunofluorescence in different groups at ×40 magnification.

**Additional file 3: Figure S2**. CD73+hucMSC-EVs reduced activation of astrocytes mainly expressed as the A1 phenotype after SCI in mice. (A and B) Changes of GFAP, C3, and S100A10, are determined by immunofluorescence in different groups. (C) Fluorescent intensities are normalized to the sham group. (*p<0.05 versus SCI group, #p<0.05 versus SCI+CD73+hucMSC-EVs group, n=5).

## Data Availability

All data are fully available without restriction.

## Abbreviations

SCI: Spinal cord injury
ATP: Adenosine triphosphate
AMP: Adenosine monophosphate
CD73: Ecto-5′-nucleotidase
MSC: Mesenchymal stem cell
hucMSC: Human umbilical cord derived mesenchymal stem cell
EVs: Extracellular vesicles
hucMSC-EV: Extracellular vesicles derived from human umbilical cord mesenchymal stem cell
CD73+ hucMSC-EV: Extracellular vesicles derived from CD73 modified hucMSCs
A2aR: Adenosine 2a receptor
A2bR: Adenosine 2b receptor
IL-4: Interleukin 4
HEK-293: Human embryonic kidney 293 T
TEM: Transmission electron microscopy
NTA: Nanoparticle tracking analysis
qRT-PCR: Quantitative real-time PCR
SPF: Specific pathogen-free
BBB: Basso-Beattie-Bresnahan
IVIS: In vivo imaging system
CSF: Cerebrospinal fluid
H&E: Haematoxylin and Eosin
TUNEL: TdT-mediated DUTP nick end labeling
DMEM: Dulbecco's Modified Eagle Medium
MSC-EV: MSC-derived extracellular vesicles
Gs: Stimulatory G alpha protein
PKA: Protein kinase A
