# Supplementary material for: Extracellular vesicles derived from CD73 modified human umbilical cord mesenchymal stem cells ameliorate inflammation after spinal cord injury
Source: J Nanobiotechnology. 2021 Sep 8;19:274. doi: 10.1186/s12951-021-01022-z (PMC8425042; doi:10.1186/s12951-021-01022-z)
Supplement: Supplementary file 1 — Additional file 1: Table S1. Sequence of the real-time PCR primers. [file 12951_2021_1022_MOESM1_ESM.docx]

**Supplemental Table 1. Sequence of the real-time PCR primers**

| **Gene** | **Sequence** |
| --- | --- |
| TNF-α | forward 5’-CCTGTAGCCCACGTCGTAG-3’ |
|  | reverse 5’-GGGAGTAGACAAGGTACAACCC-3’ |
| IL-1β | forward 5’- GCTGAAAGCTCTCCACCTCA-3’ |
|  | reverse 5’- AGGCCACAGGTATTTTGTCG-3’ |
| iNOS | forward 5’-GTTCTCAGCCCAACAATACAAGA-3’ |
|  | reverse 5’-GTGGACGGGTCGATGTCAC-3’ |
| CD86 | forward 5’-TGTTTCCGTGGAGACGCAAG-3’ |
|  | reverse 5’-TTGAGCCTTTGTAAATGGGCA-3’ |
| Arg1 | forward 5’-CTCCAAGCCAAAGTCCTTAGAG-3’ |
|  | reverse 5’-AGGAGCTGTCATTAGGGACATC-3’ |
| IL-10 | forward 5’-GCTCTTACTGACTGGCATGAG-3’ |
|  | reverse 5’-CGCAGCTCTAGGAGCATGTG-3’ |
| CD206 | forward 5’-CTCTGTTCAGCTATTGGACGC-3’ |
|  | reverse 5’-CGGAATTTCTGGGATTCAGCTTC-3’ |
| GAPDH | forward 5’- AAGGTGGTGAAGCAGGCAT-3’ |
|  | reverse 5’- GGTCCAGGGTTTCTTACTCCT-3’ |
