# Supplementary figures and images for: Extracellular vesicles derived from CD73 modified human umbilical cord mesenchymal stem cells ameliorate inflammation after spinal cord injury
Source: J Nanobiotechnology. 2021 Sep 8;19:274. doi: 10.1186/s12951-021-01022-z (PMC8425042; doi:10.1186/s12951-021-01022-z)

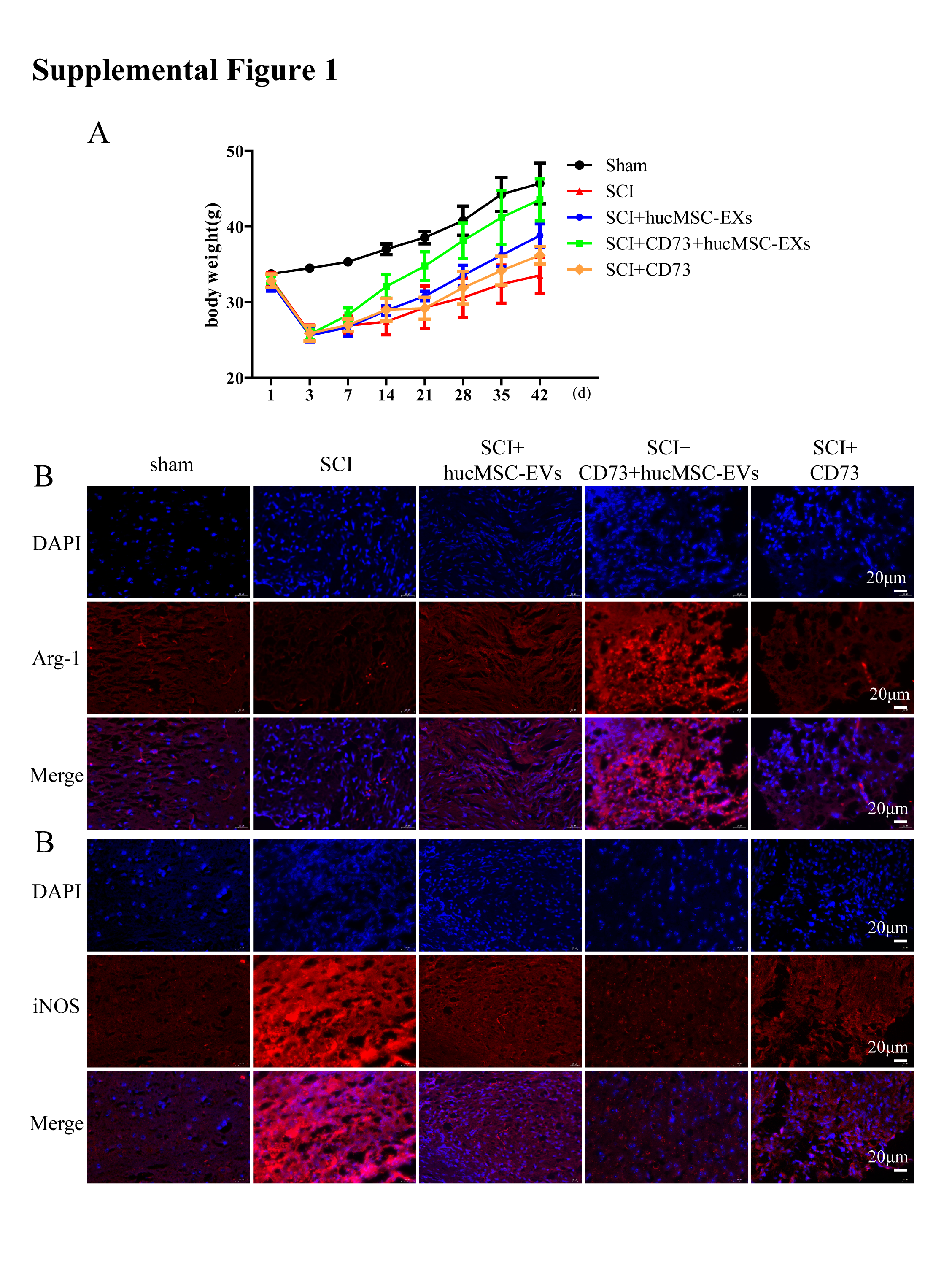

Supplement: Supplementary file 2 — Additional file 2: Figure S1. Body weights of mice and Immunofluorescence of Arginase-1 and iNOS. (A) Body weights of mice. There was a significant decrease in the body weight of all groups on day 3 after the injury. And then, mice in SCI+CD73+hucMCS-EVs group gradually recovered with a significant increase in body weight, when compared with other groups suffered from SCI. (B) Changes of arginase-1 and iNOS are determined by immunofluorescence in different groups at ×40 magnification. [file 12951_2021_1022_MOESM2_ESM.tif]

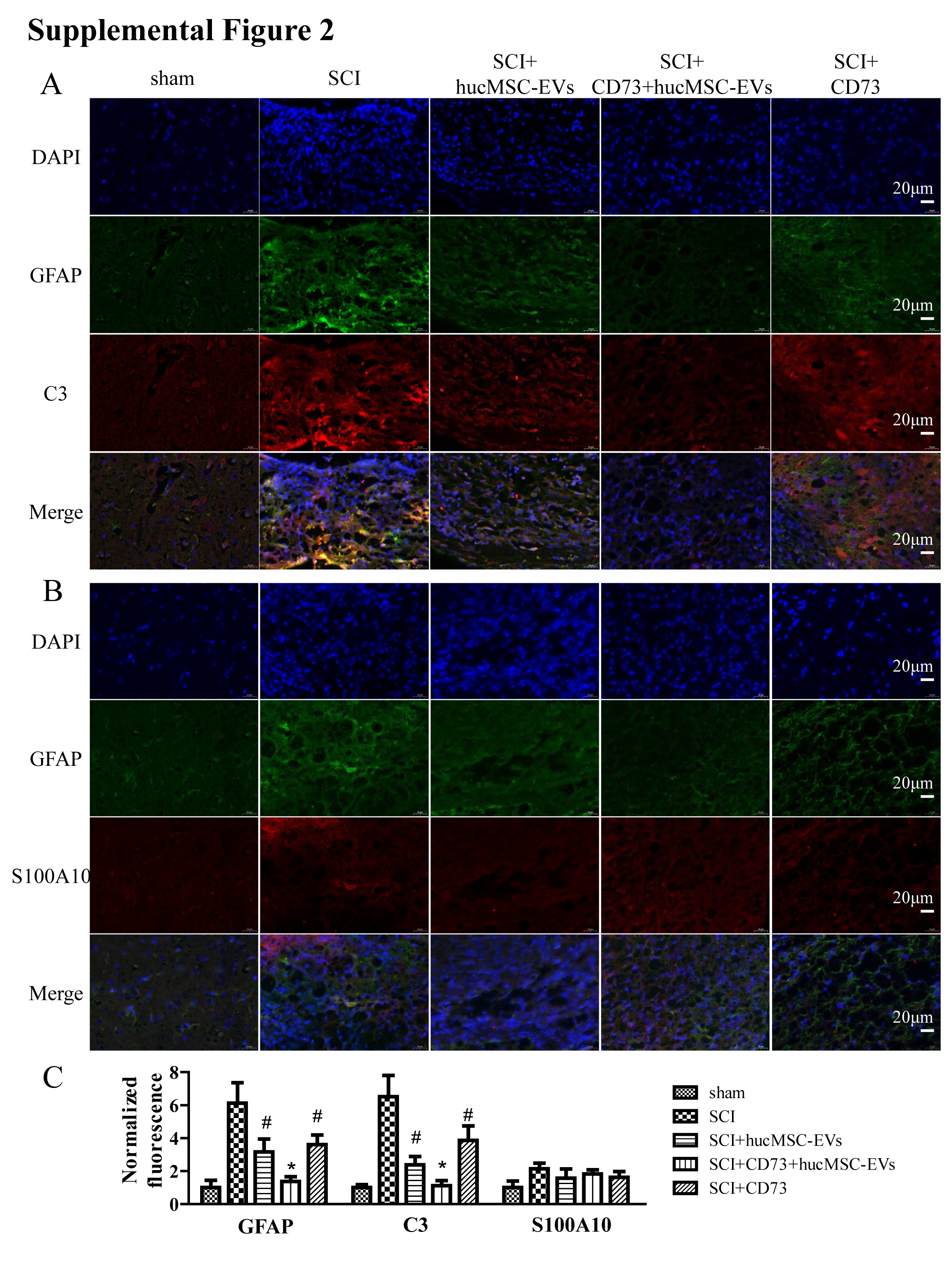

Supplement: Supplementary file 3 — Additional file 3: Figure S2. CD73+hucMSC-EVs reduced activation of astrocytes mainly expressed as the A1 phenotype after SCI in mice. (A and B) Changes of GFAP, C3, and S100A10, are determined by immunofluorescence in different groups. (C) Fluorescent intensities are normalized to the sham group. (*p<0.05 versus SCI group, #p<0.05 versus SCI+CD73+hucMSC-EVs group, n=5). [file 12951_2021_1022_MOESM3_ESM.tif]
